# HDAC5 Expression in Urothelial Carcinoma Cell Lines Inhibits Long-Term Proliferation but Can Promote Epithelial-to-Mesenchymal Transition

**DOI:** 10.3390/ijms20092135

**Published:** 2019-04-30

**Authors:** Ananda Ayyappan Jaguva Vasudevan, Michèle J. Hoffmann, Michael L. C. Beck, Gereon Poschmann, Patrick Petzsch, Constanze Wiek, Kai Stühler, Karl Köhrer, Wolfgang A. Schulz, Günter Niegisch

**Affiliations:** 1 Department of Urology, Medical Faculty, Heinrich Heine University Düsseldorf, 40225 Düsseldorf, Germany; ananda.ayyappan@med.uni-duesseldorf.de (A.A.J.V.); michele.hoffmann@hhu.de (M.J.H.); michael.beck@hhu.de (M.L.C.B.); guenter.niegisch@med.uni-duesseldorf.de (G.N.); 2 Institute for Molecular Medicine, University Hospital Düsseldorf, 40225 Düsseldorf, Germany; gereon.poschmann@hhu.de (G.P.); kai.stuehler@hhu.de (K.S.); 3 Biological and Medical Research Centre (BMFZ), Heinrich Heine University Düsseldorf, 40225 Düsseldorf, Germany; patrick.petzsch@hhu.de (P.P.); koehrer@hhu.de (K.K.); 4 Department of Otolaryngology, Medical Faculty, Heinrich Heine University Düsseldorf, 40225 Düsseldorf, Germany; constanze.wiek@hhu.de

**Keywords:** histone deacetylase, bladder cancer, HDAC4, HDAC5, transcriptomics, proteomics, EMT, epigenetics, TGFβ, TNFα

## Abstract

Class I histone deacetylases (HDACs) generally promote cell proliferation and tumorigenesis, whereas class IIA HDACs like HDAC4 and HDAC5 may promote or impede cancer development in a tissue-dependent manner. In urothelial carcinoma (UC), HDAC5 is often downregulated. Accordingly, HDAC5 was weakly expressed in UC cell lines suggesting a possible tumor-suppressive function. We therefore characterized the effects of stable HDAC5 expression in four UC cell lines (RT112, VM-Cub-1, SW1710 and UM-UC-3) with different phenotypes reflecting the heterogeneity of UC, by assessing proliferation, clonogenicity and migration ability. Further, we detailed changes in the proteome and transcriptome by immunoblotting, mass spectrometry and RNA sequencing analysis. We observed that HDAC5 overexpression in general decreased cell proliferation, but in one cell line (VM-Cub-1) induced a dramatic change from an epitheloid to a mesenchymal phenotype, i.e., epithelial-mesenchymal transition (EMT). These phenotypical changes were confirmed by comprehensive proteomics and transcriptomics analyses. In contrast to HDAC5, overexpression of HDAC4 exerted only weak effects on cell proliferation and phenotypes. We conclude that overexpression of HDAC5 may generally decrease proliferation in UC, but, intriguingly, may induce EMT on its own in certain circumstances.

## 1. Introduction

Histone deacetylases (HDACs) remove acetyl groups from histone or non-histone proteins and regulate gene expression as components of multiprotein repressor complexes [1,2,3,4]. In humans, four classes of HDACs are distinguished with overall 18 members. Among these, the class I HDACs, HDACs 1-3 and 8, are best studied in the context of cancer, where they, in general, support cell proliferation and survival [5,6]. Class IIB enzymes, especially HDAC6, appear to be particularly important for cellular stress responses. The function of the class IIA enzymes, HDACs 4, 5 and 7, in cancer is less well understood. Different from class I HDACs, they do not appear to contribute substantially to global histone acetylation and may have limited catalytic activity. Rather, they interact with transcription factors and other chromatin proteins, including class I HDACs, to repress specific genes. In addition, expression and functions of class IIA HDACs are more tissue-specific. For instance, HDAC4 and HDAC5 are important for brain development, neuronal plasticity and repair, whereas HDAC7 and HDA9 influence T-cell and B-cell development [7]. Class IIA HDACs may also be involved in cancer development, but, in contrast to class I HDACs, they may promote or impede cancer development depending on the tissue [4,7].

Our group studies HDAC function and the therapeutic potential of HDAC inhibitors in urothelial carcinoma (UC), the most common type of urinary bladder cancer. In accord with observations in many other cancers, we have previously observed that experimental downregulation or pharmacological inhibition of class I HDACs, in particular of HDAC1 and HDAC2 combined, strongly impairs proliferation and survival of UC cells [8]. The status and functions of class IIA HDACs in UC are less well understood and there are almost no dedicated studies available to date [5,9]. In the urinary bladder, HDAC4 and HDAC5 are the main class IIA HDACs according to GTex RNA expression data. In UC, genomic alterations of HDAC4 and HDAC5 are rare. According to the TCGA analysis accessed via cBioPortal [10] in February 2019, only 12 out of 411 invasive UC contained genome alterations in HDAC4, predominantly deep deletions, and seven cases contained alterations in HDAC5, predominantly missense mutations. Both genes, however, were significantly lower expressed in tumor compared to benign bladder tissues. In contrast, the sole immunohistochemical study published so far suggested more frequent upregulation of HDAC4 [11].

In a previous investigation of HDAC4 expression in UC cell lines and published microarray data sets, we found variable levels of HDAC4 protein and mRNA, respectively, in UC cell lines and tissues [12]. Overexpression of HDAC4 slightly diminished the proliferation of one UC cell line and had little effect on another one [13]. Notably, the class IIA-specific HDACi TMP-269 did not substantially impair proliferation or viability of UC cell lines, in contrast to HDAC inhibitors (HDACi) directed specifically at class I HDACs [8,13]. 

In accord with the data from the large TCGA study, we observed that HDAC5 was consistently lower expressed in UC cell lines than in normal urothelial cells. Taken together, these findings suggest a possible tumor-suppressive function of HDAC5 in UC. We therefore characterized in detail the effects of stable expression of HDAC5 on four urothelial carcinoma cell lines with different phenotypes, which reflect the heterogeneity of the disease. We observed that HDAC5 overexpression in general decreased cell proliferation, but in one cell line induced a dramatic change from an epitheloid to a mesenchymal phenotype, i.e., epithelial-mesenchymal transition (EMT). These phenotypical changes were further followed up by comprehensive proteomics and transcriptomics analyses. While HDAC5 has previously been identified as a factor in fibrosis and cancer progression, this is, to our knowledge, the first report demonstrating that HDAC5 on its own may induce EMT in certain circumstances. In accord with our previous report, overexpression of HDAC4, in comparison, had only weak effects on UC cells.

## 2. Results

### 2.1. Consistent Downregulation of HDAC5 in Urothelial Carcinoma Cell Lines

**Figure 1:**
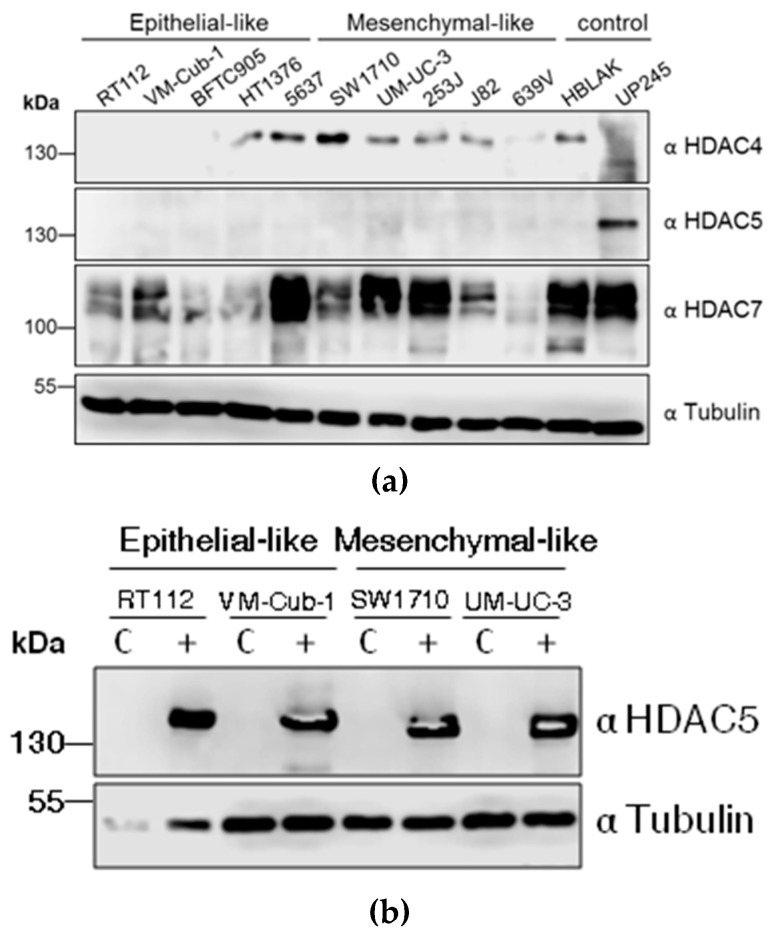
Class IIA histone deacetylases (HDAC) levels in UC cell lines (UCCs) and generation of HDAC5 stably-expressing cell lines. (**a**) Endogenous levels of HDAC4, HDAC5, and HDAC7 in the indicated UCCs and the urothelial controls HBLAK and UP245 as determined by immunoblotting of cell lysates. The description as “epithelial-like” or “mesenchymal-like” is based on morphology; see also Figure 5b. HDAC proteins were detected by the respective antibodies (α). α-Tubulin served as a loading control. (**b**) Analysis of HDAC5 overexpressing cell lines. Stably HDAC5-expressing cell lines were produced by lentivirus transduction. After selection for puromycin resistance, the cells were subjected to immunoblotting to confirm expression of HDAC5 protein. C: vector-only controls; + HDAC5-transduced cells.

To determine the endogenous protein levels of class II HDACs, HDAC4, HDAC5, and HDAC7, protein lysates from ten different UC cell lines (UCCs) and two non-cancerous controls, namely the benign urothelial cell line HBLAK and the primary urothelial culture UP245, were subjected to immunoblotting. Expression of HDAC4 and HDAC7 was variable among the cell lines. For instance, SW1710 and 5637 cells had stronger HDAC4 expression compared to the low levels in HT1376, UM-UC-3, 253J, J82, 639V and HBLAK. The expression of HDAC4 was nearly undetectable in RT112, VM-Cub-1, BFTC905, and UP245 cells. HDAC7 expression was also variable, being clearly stronger in 5637, UM-UC-3, 253J, as well as the controls HBLAK and UP245, compared to other UCCs. Notably, HDAC7 regularly appeared as a double band, which could correspond to several isoforms known to be generated by alternative splicing. In contrast to HDAC4 and HDAC7, HDAC5 levels in UCCs were uniformly low and often below the detection limit. The highest expression was observed in the normal UP245 culture (Figure 1A).

Due to its low expression, we were primarily interested in the effects of HDAC5 overexpression in UCCs. Therefore, lentiviral-based vectors were used to introduce HDAC5 into four UCCs; paired control cell lines were generated using vector only. The parental four cell lines used, RT112, VM-Cub-1, SW1710 and UM-UC-3, differ in their phenotypes with decreasing epithelial and increasing mesenchymal morphology in that order. Stable overexpression of HDAC5 was achieved in all four cell lines (Figure 1B).

### 2.2. HDAC5 Expression Impairs Long-Term Proliferation and Clonogenic Potency of UCCs

**Figure 2:**
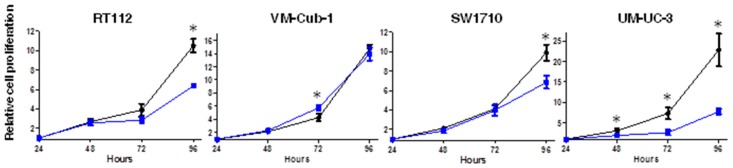
Effect of HDAC5 on cell proliferation. The cell proliferation capacity of vector-only and HDAC5-transduce cell lines was analyzed by MTT assays at 24, 48, 72, and 96 h. The 24 h value of each cell line was set as “1” at the origin of the graph. Values represent means ±  SD (error bars) of quadruplicates. Asterisks denote significant differences (*t*-test, * *p* < 0.05). Blue: HDAC5 cells; black: vector-only cells.

We next measured cell proliferation by MTT assay at different time points over 96 h. Until 72 h (48 h in the case of UM-UC-3), HDAC5 and vector-only transduced cell lines grew quite similarly, but at 96 h proliferation of HDAC5-expressing RT112, SW1710 and UM-UC-3 cells was clearly diminished (around 40%, 40%, and 70%, respectively) in comparison to their respective vector-only cells (Figure 2). Interestingly, no difference was observed between vector-only and HDAC5-expressing VM-Cub-1 cells over the entire period (Figure 2). These results were validated by direct counting of total and viable cells by flow cytometry (Supplementary Figure S1).

**Figure 3:**
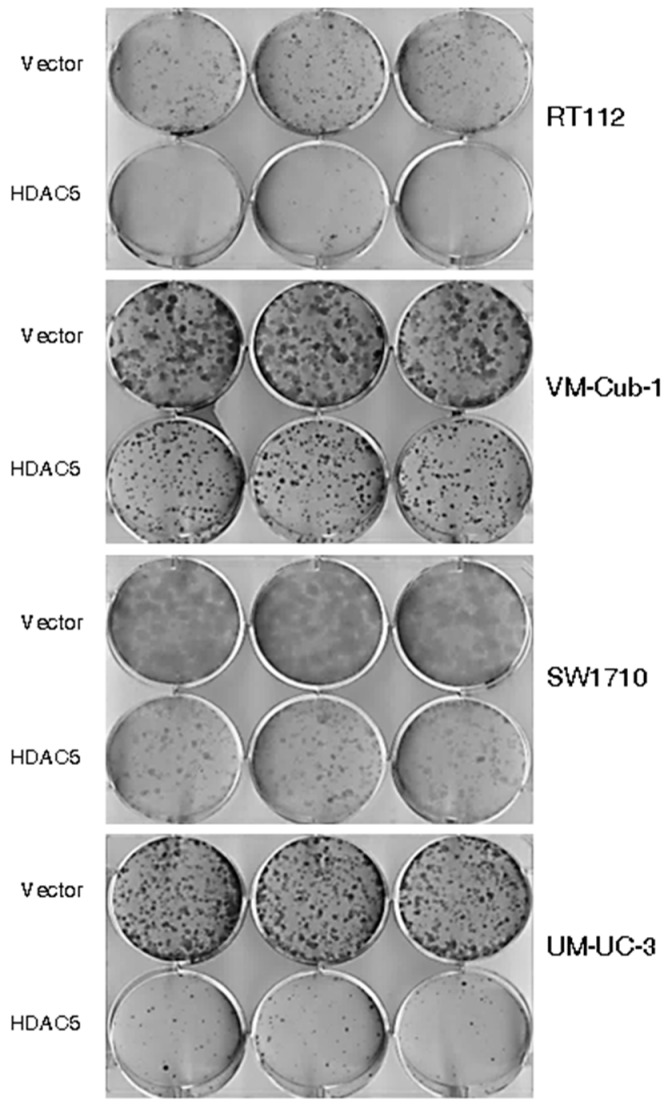
Effect of HDAC5 on clone formation. Representative pictures of clone formation assays after seeding of equal numbers of cells from the indicated vector-only or HDAC5-transduced UCCs.

**Figure 4:**
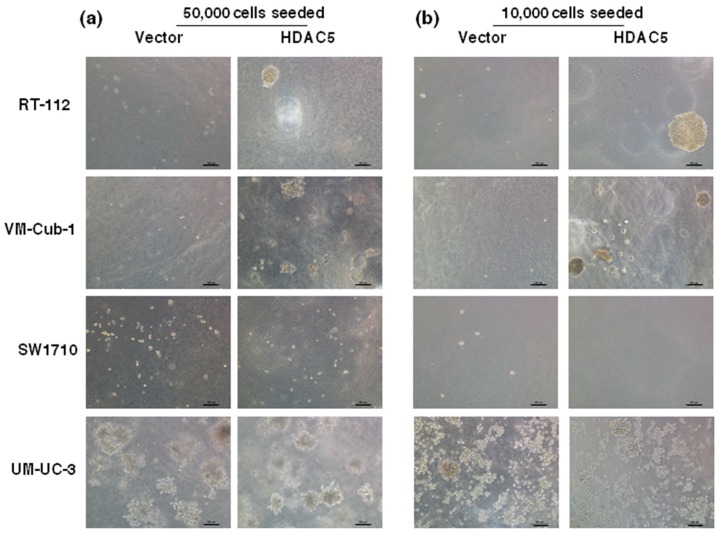
Colony formation of vector-only and HDAC5-transduced cells in soft agar. Soft agar colony formation assays were performed by seeding 50,000 cells (**a**) and 10,000 cells (**b**). Several images were captured and representative pictures for each cell variant are shown. The scale bars are 100 µm.

The decrease in proliferative ability over time conferred by HDAC5 was also reflected in clone formation assays. The ability to form clones following seeding at low density in tissue culture plates was strongly diminished in HDAC5-transduced RT112, SW1710 as well as UM-UC-3 cells, and to a lesser extent in VM-Cub-1, compared to their respective vector-only controls (Figure 3). Upon seeding in soft agar, UM-UC-3 HDAC5-transduced cells formed smaller clones than their vector controls, whereas neither variant of SW1710 formed large colonies. Strikingly, however, HDAC5-transduced RT112 and VM-Cub-1 cells acquired the ability to form colonies in soft agar, which the parental cells and the vector-only controls lack (Figure 4). Notably, HDAC5 expressing VM-Cub-1 formed loose aggregates, whereas HDAC5 expressing RT112 cells were compact and bigger, but fewer in number (Figure 4).

### 2.3. HDAC5 Induces an Epithelial-Mesenchymal Transition in VM-Cub-1 Cells

**Figure 5:**
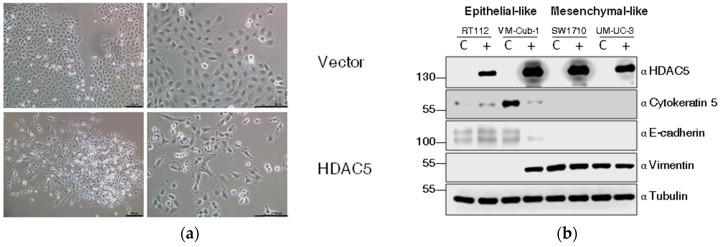

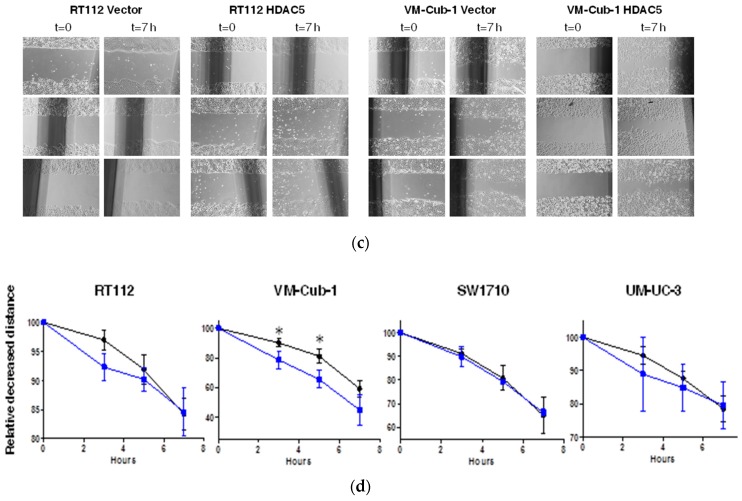
HDAC5 triggers an epithelial-mesenchymal transition in VM-Cub-1. (**a**) Cell morphology of VM-Cub-1 vector and HDAC-5 cells was analyzed by microscopy, images were captured at different magnifications. The scale bars are 100 µm. (**b**) Equal amount of proteins from vector and HDAC5 expressing cells were subjected to immunoblotting. Cytokeratin 5 and E-Cadherin served as an epithelial marker and Vimentin as a mesenchymal marker. “α” denotes “antibody. C: vector-only, + HDAC5-transduced cells. (**c**) Results of migration assays. Representative images of cells at 0 h and 7 h. (**d**) Evaluation of migration assays. The distance at 0 h of each cell line was set as “100” and the decreasing lengths between the cell fronts were additionally measured after 3, 5 and 7 h. Values represent means ±  SD (error bars) of triplicates. Asterisks denote significant differences (t-test, * *p* < 0.05). Blue: HDAC5-transduced cells; black: vector-only cells.

Among UCCs, almost exclusively, cell lines with a more mesenchymal morphology form colonies in soft agar. Accordingly, the morphology of HDAC5-transduced VM-Cub-1 cells changed towards a more mesenchymal morphology and the cells grew in a more dispersed pattern rather than as tight colonies (Figure 5a). 

We therefore investigated markers of epithelial-mesenchymal transition by immunoblotting. Indeed, in VM-Cub-1 HDAC5-transduced cells, the amounts of the epithelial markers Cytokeratin 5 and E-Cadherin were diminished compared to the control, and the expression of the mesenchymal marker Vimentin was increased to a similar level as in SW1710 and UM-UC-3 cells (Figure 5b). In the other UCCs, none of these markers underwent a major change and gross morphologies appeared unaltered.

Since a more mesenchymal phenotype is often associated with increased migratory ability, we compared HDAC5-transduced to vector-only transduced UCCs in cell migration assays. A clear increase in migration was seen for HDAC5-expressing VM-Cub-1 cells over the entire duration of the experiment, whereas no significant difference in migration velocity was observed among vector-only and HDAC5-transduced SW1710 cells. RT112 and UM-UC-3 cells appeared to migrate slightly faster at earlier time points, but the differences were not statistically significant (Figure 5c). 

### 2.4. The Proteome of VM-Cub-1 Cells is Profoundly Altered by HDAC5

**Figure 6:**
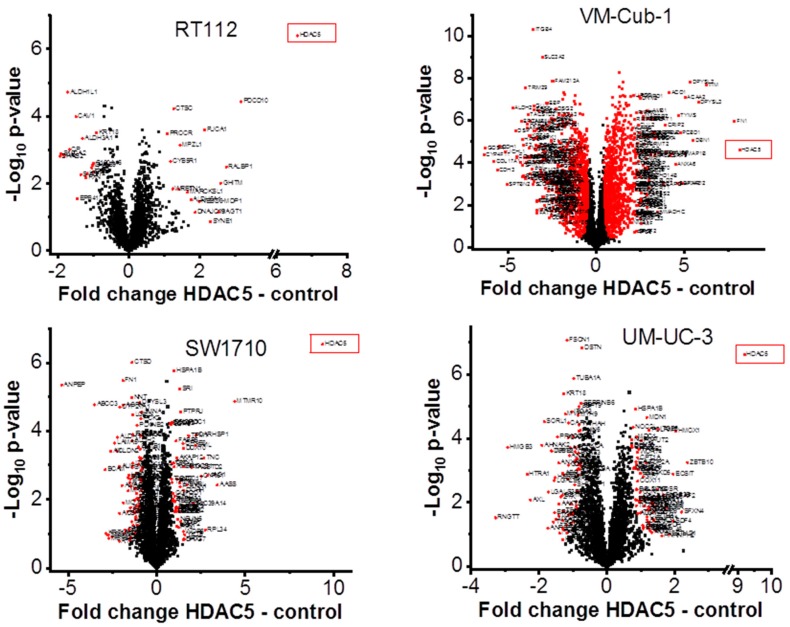
Volcano plot analysis of proteomic changes in UC cell lines transduced with vector only or HDAC5. Significantly differentially abundant proteins in HDAC5 overexpressing cells are marked by red squares. Gene names for VM-Cub-1 are shown only for proteins with at last a two-fold change. The indicated fold changes “HDAC5—control” represent the difference of mean log2 transformed intensities of HDAC5 and vector samples.

**Figure 7:**
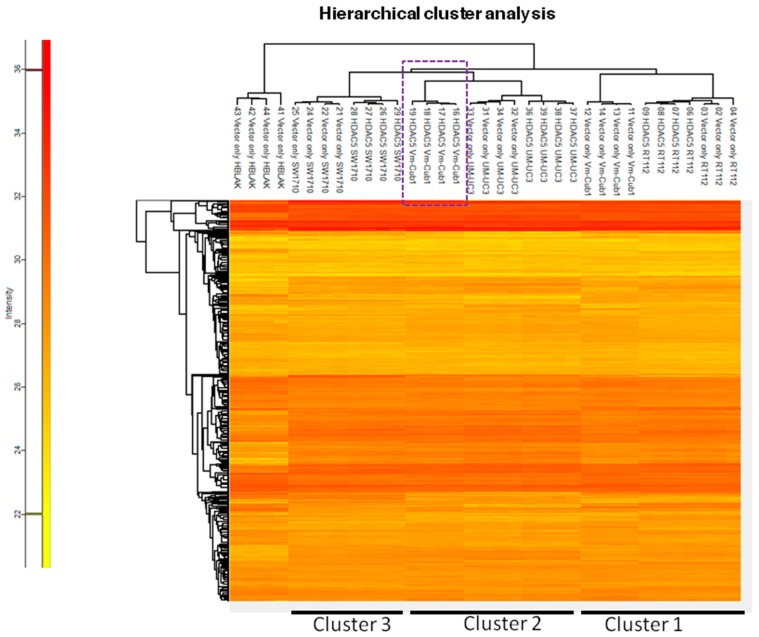
Hierarchical cluster analysis of proteome changes in UC cell lines transduced with vector only or HDAC5. HBLAK cells transduced with vector only were used as an outlier reference in this analysis.

To characterize the overall changes in the proteome of the UCCs following HDAC5 overexpression, we performed high-throughput proteomics analysis by mass spectrometry. In accord with the morphological changes, by far the most profound alteration of the proteome was observed in VM-Cub1 cells where more than half of the quantified proteins (1747 proteins) were significantly differently abundant in HDAC5-transduced versus vector-only cells. In comparison, a moderate number of significant changes were observed in SW1710 (145 proteins), and UM-UC-3 cells (154 proteins), and comparatively few in RT112 (33 proteins) (Figure 6). Hierarchical cluster analysis of the data revealed three clusters. The first cluster encompassed the epithelial RT112 vector, RT112 HDAC5, and VM-Cub-1 vector cells. The second prominent cluster consisted of vector and HDAC5 UM-UC-3 cells, and VM-Cub-1 cells transduced with HDAC5. The third cluster was made up of SW1710 vector as well as HDAC5-transduced cells. The proteome of HBLAK cells was included in this analysis, and these benign urothelial cells clustered apart from the UCCs, as expected (Figure 7). 

**Figure 8:**
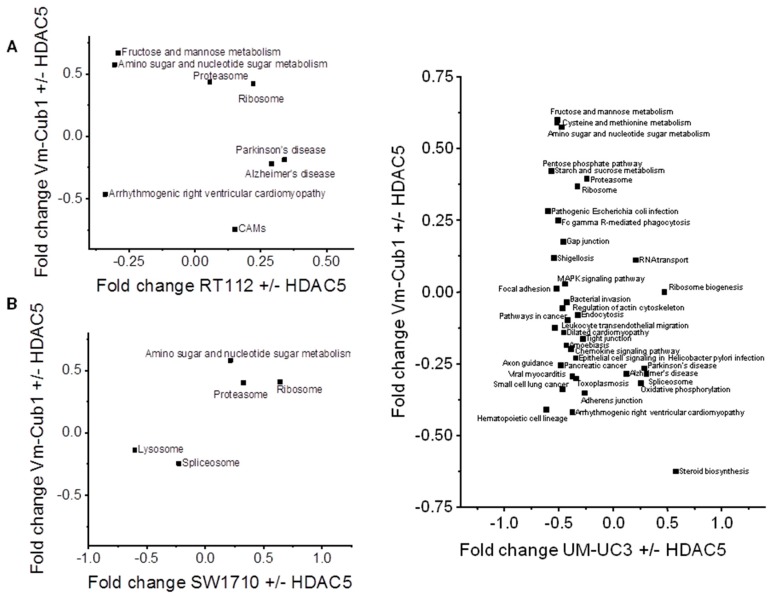
2D annotation analysis of Kyoto Encyclopedia of Genes and Genomes (KEGG) pathways in HDAC5-transduced UCCs comparing changes in VM-Cub-1 cells to those in the other indicated cell lines.

Finally, we searched for pathways that might underlie the different reaction of VM-Cub-1 to HDAC5 overexpression compared to the other cell lines. KEGG (Kyoto Encyclopedia of Genes and Genomes) pathway analysis identified metabolic processes, especially “amino sugar and nucleotide sugar metabolism”, as well as “proteasome” and “ribosome” as consistently enriched in VM-Cub-1 compared to the other three UC cell lines (Figure 8). The changes in the “amino sugar and nucleotide sugar metabolism” pathway appear to reflect in particular altered metabolism of N-acetylglucosamine, which has previously been linked to HDAC4 function in the heart [14]. Both HDAC4 and HDAC5 are established as regulators of glucose metabolism [15]. Conversely, no KEGG pathway was consistently enriched in the other UCCs compared to VM-Cub-1.

### 2.5. Transcriptome Changes by HDAC5 Are Most Extensive in VM-Cub-1 and Hint at an Involvement of TGFβ

**Figure 9:**
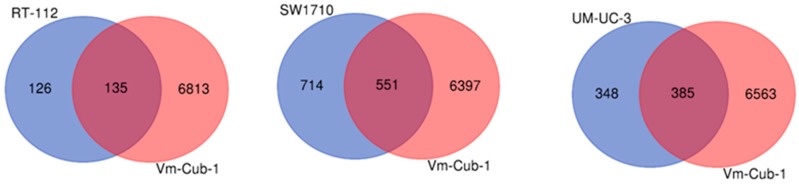
Venn diagram comparison of differentially expressed genes in HDAC5-transduced cell lines. Pairwise comparison of differentially regulated genes in RT112, SW1710, UM-UC-3 (from left to right, respectively) vs. VM-Cub-1.

Like mass spectrometry, RNA sequencing analysis revealed the most pronounced changes in the transcriptome of VM-Cub-1. In this cell line, expression of 6948 genes was significantly changed, considering Bonferroni adjustment with *p* ≤ 0.05 and at least two-fold differences. As in the proteome analysis, more moderate numbers of genes were differentially expressed in SW1710 and UM-UC-3, namely 1,265 and 733 genes, respectively. Likewise, the lowest number of changes was observed in RT112, with 261 genes differentially expressed (Figure 9, Table 1). Top canonical pathways identified by Ingenuity Pathway analysis (IPA) (Table 2) included axonal guidance, a process well-known to be influenced by HDAC5 [16] and hepatic fibrosis (see Discussion). Top upstream regulators identified by IPA included TNFα (tumor necrosis factor α), several steroid or steroid-related hormones, and, most intriguingly, TGFβ (transforming growth factor β) (Table 3). However, their activation states were predicted to be not influenced uniformly into the same direction among the four cell lines. We were most interested in TGFβ, as this factor could explain several phenotypes observed in the HDAC5-transduced cells. TGFβ inhibits the proliferation of many epithelial cells, can induce EMT in some, and was actually discovered as one factor that confers the ability for anchorage-independent growth in soft agar, together with the EGF-like TGFα [17]. We therefore speculated that mimicking of TGFβ action might explain the most prominent effects of HDAC5 on VM-Cub-1 and the other UC cell lines.

### 2.6. Effects of Added TGFβ and TNFα on Epithelial UC Cell Lines Are Not Influenced by HDAC5

**Figure 10:**
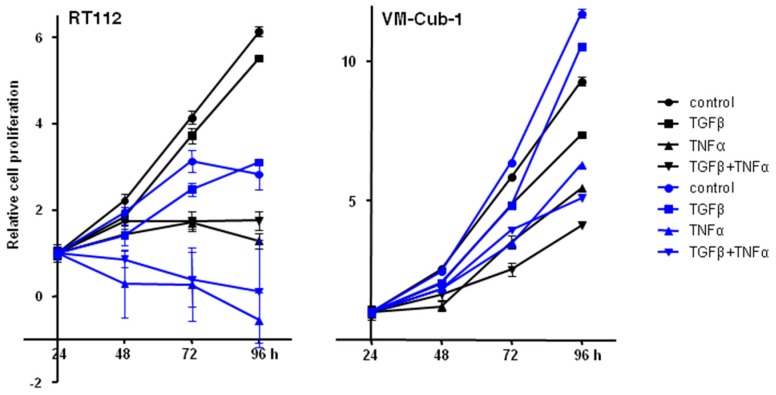
Effect of TGFβ, TNFα, or concomitant TGFβ + TNFα treatments on vector-only and HDAC5 expressing UCCs. Cells were treated with TGFβ, TNFα, or both, or left untreated. Cell proliferation was followed by MTT assays at 24, 48, 72, and 96 h. The 24 h data of each cell line was set as “1”. Values represent means ±  SD (error bars) of quadruplicates. left: RT112, right: VM-Cub-1 cells. Black lines and symbols: vector-only cells; Blue lines and symbols: HDAC5 transduced cells. All cytokine treatments caused significant (*t*-test, *p* < 0.05) decreases compared to the respective untreated controls, except for the TGFβ treatment of RT112 at 96 h.

We thus wondered whether overexpression of HDAC5 might modulate the effects of TGFβ and TNFα on UC cell lines, in particular, on the two epithelial cell lines RT112 and VM-Cub-1. In both vector-only transduced cell lines, TNFα inhibited proliferation significantly, and was clearly cytotoxic in RT112, whereas the inhibitory effects of TGFβ were slight by comparison (Figure 10). At most additive effects were achieved by the combination of the two cytokines. None of the effects of the cytokines were different in HDAC5-transduced cells, if the effects of HDAC5 per se are taken into account.

No indications of EMT were observed by microscopic inspection upon treatment of vector-only or HDAC5-transduced RT112 or VM-Cub-1 cells with TGFβ or TNFα, or their combination. This observation was substantiated by immunoblot analysis for the EMT markers E-Cadherin and Vimentin, which were not affected by the cytokine treatments (Supplementary Figure S2).

### 2.7. Weak Effects of HDAC4 Overexpression on UC Cell Lines 

In parallel experiments, we investigated the effect of HDAC4 overexpression on UC cell lines. Notably, unlike HDAC5, HDAC4 is not uniformly downregulated in UC cell lines. Rather, expression ranges widely, with some UC cell lines expressing higher, but others lower HDAC4 protein levels compared to normal control cells ([12] and Figure 1). Also, in preliminary experiments on two cell lines, we had observed very modest effects of HDAC4 overexpression on cell growth, especially in the short-term [13].

**Figure 11:**
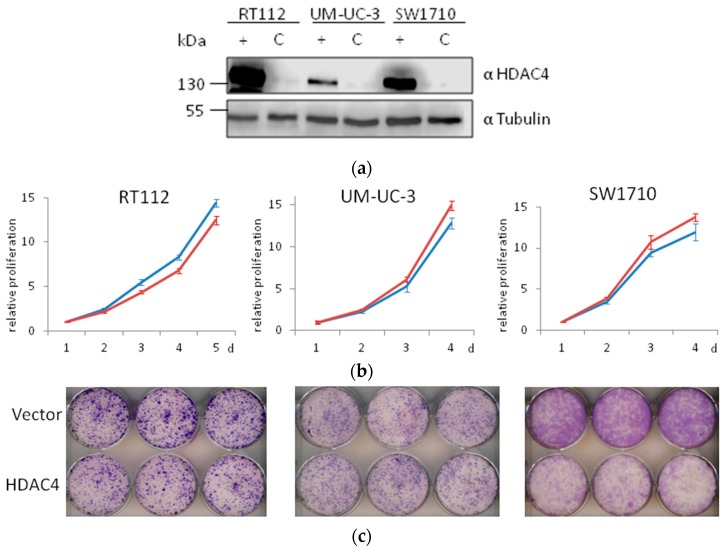
Effects of HDAC4 overexpression on UC cell lines. (**a**) Immunoblot validation of HDAC4 overexpression in three UC cell lines. α-Tubulin was used as a loading control. C: vector-only, + HDAC4-transduced cells. (**b**) MTT assay of proliferation of vector-only (red) or HDAC4-transduced (blue) cell lines. For each cell line, control values at day 1 are set as 1. (**c**) Representative photographs of clone formation assays of vector-only (top) or HDAC4-transduced (bottom) cell lines.

Here, we generated three more HDAC4-overexpressing UC lines by lentiviral transduction. Overexpression of HDAC4 was successfully achieved (Figure 11a). In short term experiments, no significant differences were observed in the growth of vector-only and HDAC4-transduced cell line pairs (Figure 11b). In clone formation assays, differences were apparent, however—as in our previous series of experiments—were more modest than those in HDAC5-transduced cell lines (Figure 11c). No morphological changes were observed. Specifically, HDAC4-overexpressing VM-Cub-1 cells did not undergo EMT [13], like their vector-only transduced and parental counterparts.

Taken together, overexpression of HDAC4 compromised the growth ability of UC cell lines, if at all, to a much lower extent than overexpression of HDAC5, and did not induce detectable morphological changes.

## 3. Discussion

Class I HDACs are firmly established as important factors in the proliferation of normal cells and various cancers and consequently, as suitable targets for HDAC inhibitors in cancer treatment [3,5,6,9]. By comparison, class IIA HDACs are best known for their involvement in the development and functions of specific tissues and their relevance in the context of cancer is not well investigated. In urothelial carcinoma, in particular, the importance of class I HDACs, especially of HDAC1 and HDAC2, has been underlined by recent dedicated studies, in keeping with general thought (reviewed in references [5,9]). Whereas these HDACs are rather upregulated in UC, HDAC4 and HDAC5 are often downregulated at the mRNA level. Our former analyses of published RNA microarray data and protein expression in UC cell lines had indicated a high variability of HDAC4 among the cases [12], as confirmed here. In contrast, we found HDAC5 consistently down-regulated in UCCs suggesting that its presence might impede their growth.

Lentiviral transduction of HDAC5 indeed decreased the short-term proliferation of three of the four UCCs and their ability to form clones. Of note, these three cell lines cover a large spectrum of the disease, ranging from a luminal, rather well-differentiated (RT112) to an aggressive, undifferentiated and mesenchymal cell line (UM-UC-3). The most interesting case was however the VM-Cub-1 cell line, which is considered as representative of the basal subtype and moderately differentiated. This cell line was not significantly impeded in its growth, but underwent a dramatic phenotypic change with obvious EMT and acquisition of the ability for anchorage-independent growth. 

The different response to HDAC5 between VM-Cub-1 cells and the other cell lines was not only visible morphologically, but was also reflected in the proteome and transcriptome of this cell line. Compared to the other cell lines, changes in VM-Cub-1 were more numerous and more pronounced. It is not clear why VM-Cub-1 reacts differently from the other UCCs to HDAC5. Evidently, SW1710 and particularly UM-UC-3 already possess mesenchymal morphology in the absence of HDAC5. RT112, on the other hand, is more strictly epithelial and grows in tighter clusters than VM-Cub-1. Indeed, we have observed in a previous study that VM-Cub-1 cells but not 5637, another strictly epithelial cell line, underwent EMT in response to overexpression of the lncRNA HOTAIR [18]. Interestingly, indicators of EMT are also observed in selected UC in vivo, including some cancers progressing from the basal-squamous molecular subtype, to which VM-Cub-1 belongs [19]. Our observation thus may reflect that certain UC, like VM-Cub-1, are more prone to induction of EMT.

It is indeed intriguing that expression of HDAC5 on its own is capable of inducing EMT in certain circumstances, and notably, appears to be more effective than even TGFβ, which can promote enhanced migration and invasion in some UCCs [20]. While the involvement of HDAC5 in EMT is overall poorly studied to date, in accord with our findings, upregulation of HDAC5 was reported to promote EMT in non-small cell lung cancer cells [21]. Moreover, HDAC5 and HDAC4 were both upregulated in a model of renal fibrosis and suggested to contribute to its pathogenesis [22]. Major regulators of renal fibrosis are TGFβ [23] and TNFα [24]. Indeed, analysis of transcriptomics data from the HDAC5-transduced UC cell lines by IPA highlighted parallels between the effects of HDAC5, hepatic fibrosis, TGFβ and TNFα signaling. We therefore investigated whether the presence of HDAC5 would change the response of the UC cell lines to the two cytokines. However, the growth-inhibitory effects of the cytokines were not accentuated and—at least across the time frame investigated—neither TGFβ nor TNFα induced EMT markers, with or without HDAC5. Thus, HDAC5 shares targets with the two cytokines, but does not appear to modify their action in UC cells.

Obviously, overexpression of any protein in a cell line has the potential to cause unspecific effects irrelevant to its normal biological function. This is also a concern in our study. However, the comparison between the effects of HDAC4, which were modest at most, and those of HDAC5, which were prominent, indicates a high degree of specificity and suggests that the effects observed with HDAC5 overexpression reflect biologically relevant actions.

In conclusion, according to global gene expression analyses, the class IIA HDAC4 and HDAC5 are both lower expressed in urothelial carcinoma than in normal bladder tissue. In UC cell lines, however, HDAC5, but not HDAC4 is consistently weakly expressed. Accordingly, lentiviral transduction of HDAC5, but not HDAC4 had significant effects on cellular properties. Supporting a tumor-suppressive function of HDAC5 in UC, its overexpression diminished growth in three out of four cell lines. However, induction of EMT in a fourth cell line hints at a potential of HDAC5 to promote a more aggressive phenotype in individual cases. Strikingly, we found HDAC5 overexpression on its own can be capable of inducing EMT. Our findings call for further analyses of the relation between HDAC5 on the one hand, and EMT and fibrosis, on the other hand, in cancer and other diseases.

Finally, the overall aim of our project is defining an optimal HDAC target profile for HDAC inhibitors for the treatment of UC [9]. In this regard, our present findings further support the conclusion that targeting class IIA HDACs in this tumor may rather be counterproductive.

## 4. Materials and Methods 

### 4.1. Cell Culture 

The urothelial cancer cell lines (UCCs) VM-Cub1, RT112, SW1710, and UM-UC-3 were provided by Dr. M. A. Knowles (Leeds, UK), Dr. J. Fogh (New York, NY, USA) and Dr. B. Grossmann (Houston, TX, USA) or by the DSMZ (Braunschweig, Germany). They were cultured in DMEM GlutaMAX-I (Gibco, Life Technologies, Darmstadt, Germany) supplemented with 10% fetal calf serum (Biochrom, Berlin, Germany) at 37 °C in a humidified atmosphere of 5% CO_2_. As a benign urothelial control, we used the HBLAK cell line ([25], spontaneously immortalized from primary human bladder epithelial cells; obtained from CELLnTEC, Bern, Switzerland), which were cultured in CnT-Prime Epithelial Culture Medium (CELLnTEC, Bern, Switzerland). All cell lines were authenticated by DNA fingerprint analysis. Normal urothelial cells (UP) were cultured as described [25] with informed consent of the donors and approval by the Ethics Committee of the Medical Faculty of the Heinrich-Heine-University, study number 1788.

Cytokines TGFβ (Human, recombinant TGF-β1, Miltenyi Biotec, Bergisch Gladbach, Germany, reconstituted in water) and TNFα (Human, recombinant TNFα, Sigma-Aldrich, St. Louis, MO, USA, dissolved in phosphate buffered saline (PBS)) were used at 5 ng/mL and 20 ng/mL, respectively.

### 4.2. Generation of Stably Eexpressing HDAC5 and Vector Control UC Cell Lines 

The plasmid pcDNA3.1+HDAC5-FLAG was a kind gift from Eric Verdin (Addgene plasmid #13822) [1]. The HDAC5 open reading frame with a C-terminal FLAG tag was subcloned into the lentiviral transfer vector puc2CL12IPwo using primers forward 5′-CATCTCGAGGCCACCATGCCCAGTTCCATGGG and reverse 5′-ATCGCTAGCTTActtgtcatcgtcgtccttgtagtcTCCTCCCAGGGCAGGCTCCTGC. The construct was verified by Sanger sequencing. The HDAC4 lentiviral vector has been described [13]. Lentivirus production and cell transduction were performed as previously described [13,26]. Briefly, HEK-293T cells were transfected with helper plasmid expression construct pCD/NL-BH, envelope vector (pczVSV-G) and either the vector plasmids puc2CL12IPwo or puc2CL12IPwo-HDAC5-FLAG. Replication-deficient lentiviral particles were harvested 48 h after transfection and used to transduce RT112, VM-Cub-1, SW1710, and UM-UC-3 cells using 8 µg/mL polybrene (Sigma-Aldrich). Twenty-four hours after transduction, the supernatant containing viral particles was removed and the transduced cells were selected and maintained with 1 µg/mL puromycin (Invitrogen, Carlsbad, CA, USA). Stable expression of HDAC5 was confirmed by immunoblot analysis. 

### 4.3. Clone Formation Assays 

Colony forming potency of vector and HDAC5 expressing UCCs was assessed by Giemsa staining. One thousand cells (in triplicates) were seeded in 6 well plates and cultured for 8 d or 10 d, for RT112, until macroscopically visible colonies had developed. Then, colonies were washed with PBS, fixed in methanol and stained with Giemsa (Merck Millipore, Darmstadt, Germany).

Soft agar assays were performed by a standard method [27] in DMEM with 10% fetal calf serum.

### 4.4. Cell Proliferation Analysis

Cell viability as an indicator of proliferation was determined by MTT (3-(4,5-dimethylthiazol-2-yl)-2,5-diphenyltetrazolium bromide, Sigma-Aldrich) assays as described [28]. One thousand cells per well were seeded in 96-well plates. After the incubation period, 10 µL MTT stock solution (5 mg/mL in PBS) was added per well and incubated for 60 min at 37 °C. The medium was discarded and the formazan product was dissolved in 50 µL DMSO. Absorbance was analyzed spectrophotometrically at 550 nm against the reference wavelength of 750 nm. Wells containing medium only served as a blank.

### 4.5. Migration Assay 

Vector-only and HDAC5-transduced UCCs (3 × 10^4^ cells/well) were seeded around Ibidi inserts in 50 µl medium on either side. The next day, inserts were detached to form a gap between the two sections. Cells were once gently washed with media and left to migrate for up to 24 h. Images were taken at the indicated time points and cell migration was measured as the distance between opposite cell fronts and plotted. Measurements were performed in triplicates. 

### 4.6. Counting of Viable Cells by Flow Cytometry

For cell counting, cells were trypsinized and resuspended in PBS. Shortly before analysis, 1 µg/mL propidium iodide was added. Cells excluding the stain and total cell numbers were then counted in a MACSQuant Analyzer (Miltenyi Biotech, Bergisch Gladbach, Germany) and MACSQuantify software as previously described [8].

### 4.7. Immunoblot Analysis

Immunoblot analysis of whole cell extracts was performed as described [28]. Cells were lysed in radioimmunoprecipitation assay (RIPA)-buffer (150 mM NaCl, 1% Triton X-100, 0.5% deoxycholate, 1% Nonidet P-40, 0.1% SDS, 1 mM EDTA, 50 mM TRIS, pH 7.6), containing a protease inhibitor cocktail (10 µl/mL, #P-8340, Sigma-Aldrich). Lysates were clarified by centrifugation (20 min, 14,800 rpm, 4 °C) and protein concentration was determined by BCA assay. Twenty micrograms of each protein lysate with 6× SDS sample buffer were boiled at 95⁰C for 5 min and subjected to SDS-PAGE followed by transfer onto PVDF membrane (Merck Millipore, Schwalbach, Germany) by electroblotting. Membranes were blocked with skimmed milk solution prepared in TBST (150 mM NaCl, 10 mM TRIS, pH 7.4 and 0.1% Tween-20) and probed with the following primary antibodies: mouse anti-HDAC4 antibody (1:500 dilution, sc-46672, Santa Cruz biotechnology, Heidelberg, Germany); goat anti-HDAC5 antibody (1:1000 dilution, sc-5250, Santa Cruz biotechnology); mouse anti-HDAC7 antibody (1:1000 dilution, sc-74563, Santa Cruz biotechnology); rabbit anti-Vimentin antibody (1:1000 dilution, ab92547, abcam, Berlin, Germany); rabbit anti-E-Cadherin antibody (1:1000 dilution, #3195, Cell Signaling technology, Frankfurt, Germany); rabbit anti-Cytokeratin 5 antibody (1:1000 dilution, ab53121, abcam); mouse anti-α-tubulin antibody (1:50,000 dilution, clone B512, Sigma-Aldrich). Secondary antibodies were horseradish peroxidase-conjugated goat-anti-mouse antibody (sc-2005, Santa Cruz biotechnology), goat-anti-rabbit antibody (sc-2004, Santa Cruz Biotechnology) and rabbit-anti-goat antibody (P0160; DakoCytomation, Stockholm, Sweden) at dilutions of 1:10,000 to 1:100,000. Signals were visualized by SuperSignal™ West Femto (Thermo Scientific, Rockford, IL) or WesternBright Quantum kit (Biozym, Hessisch Oldendorf, Germany).

### 4.8. RNA Isolation, Sample Preparation, and High Throughput mRNA Sequencing 

Cells were harvested with Qiazol (Qiagen, Hilden, Germany) and lysates were stored at −80 °C. Total RNA was then isolated by the Qiagen RNeasy Mini Kit (Qiagen, Hilden, Germany). RNA quality was checked by spectrophotometry. 

### 4.9. Proteome Analysis by Label-Free Quantification Based Mass Spectrometry

To study the effect of HDAC5 overexpression on selected cell lines, quadruplicates from individual culture dishes were prepared from RT112, VM-Cub-1, SW1710, and UM-UC-3 cells expressing HDAC5 as well as corresponding and HBLAK vector-only cells. Cells were harvested and protein lysates prepared in an aqueous urea containing buffer (2 M thiourea, 7 M urea, 4% (*w*/*v*) 3-[(3-Cholamidopropyl)dimethylammonio]-1-propanesulfonate, 30 mM Tris-HCl, pH 8.0) and prepared for mass spectrometric analysis, as described elsewhere [29]. Briefly, proteins were stacked in an acrylamide gel (about 4 mm running distance), subjected to silver staining, de-stained, reduced and alkylated and digested with trypsin. Resulting peptides were extracted from the gel and 500 ng peptides prepared in 0.1% trifluoroacetic acid for liquid chromatography and mass spectrometric analysis.

Here, first peptides were separated by liquid chromatography. An Ultimate 3000 Rapid Separation liquid chromatography system was used for peptide separation over a two hour gradient before analyzing peptides with a QExactive plus mass spectrometer in data dependent top ten mode essentially as described [29].

For spectra identification and precursor ion intensity based quantification, the MaxQuant environment (version 1.6.0.16, MPI for Biochemistry, Planegg, Germany) was used with standard parameters. Spectra were matched against sequence data from the Homo sapiens proteome (UniProt proteome 5640, 71567 entries, downloaded on 28th August 2017). Further search parameters and parameters for peptide and protein acceptance and quantification were essentially as described previously [29].

Further analysis of quantitative data was carried out with Perseus (version 1.6.0.7, MPI for Biochemistry, Planegg, Germany) within the R environment (R foundation for statistical computing). Here, only proteins showing at least two different peptides were considered and proteins showing at least three valid quantitative values in at least one group in the respective comparison. For the identification of HDAC5 affected proteins and differences between the cell lines, a two way ANOVA followed by a Benjamini Hochberg correction and by Tukey’s honest significance tests was carried out on log2 label-free quantification intensities after missing values were filled in with random values from a normal distribution (width: 0.3 standard deviations, downshift: 1.8 standard deviations). Additionally, Student’s t-test were calculated for pairwise comparisons of HDAC5-transduced and vector-only cells and cutoffs were determined by the significance analysis of microarrays method (S0 = 0.8, 5% false discovery rate). 

Differences of the mean values of log2 label-free quantification intensities were further used for categorical annotation enrichment analyses.

### 4.10. RNA-Seq and Data Analysis

Total RNA samples used for transcriptome analyses were quantified (Qubit RNA HS Assay, Thermo Fisher Scientific) and quality measured by capillary electrophoresis using the Fragment Analyzer and the Total RNA Standard Sensitivity Assay (Agilent Technologies, Santa Clara, CA, USA). All samples in this study showed high quality RNA Quality Numbers (RQN; mean = 9.8). The library preparation was performed according to the manufacturer’s protocol using the ‘TruSeq Stranded mRNA Library Prep Kit’ from Illumina®. Briefly, 250 ng total RNA were used for mRNA capturing, fragmentation, the synthesis of cDNA, adapter ligation and library amplification. Bead purified libraries were normalized and finally sequenced on the HiSeq 3000/4000 system (Illumina, San Diego, CA, USA) with a read setup of 1x150 bp. The bcl2fastq tool was used to convert the bcl files to fastq files as well for adapter trimming and demultiplexing. 

Data analyses on fastq files were conducted with CLC Genomics Workbench (version 10.1.1, QIAGEN, Venlo, Netherlands). The reads of all probes were adapter trimmed (Illumina TruSeq) and quality trimmed (using the default parameters: bases below Q13 were trimmed from the end of the reads, ambiguous nucleotides maximal 2). Mapping was done against the Homo sapiens (hg38) (Mai 25, 2017) genome sequence. After grouping of samples (four biological replicates each, except for three for RT112) according to their respective experimental condition, multi-group comparisons were made and statistically determined using the Empirical Analysis of DGE (version 1.1, cutoff = 5). The Resulting P values were corrected for multiple testing by FDR and Bonferroni-correction. A P value of ≤0.05 was considered significant.

RNA-Seq data were further analyzed with the Ingenuity Pathway analysis software (Qiagen, Hilden, Germany) using the expression data with p(FDR) ≤ 0.05 and fold-change ≥ 1.7. The number of gene hits with this filter were 606 (RT112), 8000 (VM-Cub-1), 2419 (SW1710), and 1527 (UM-UC-3) in HDAC5-expressing cells compared to their vectors.

### 4.11. Statistical Analysis 

Data are represented as the mean with standard deviation (SD). Statistically significant differences between two groups were analyzed using the unpaired Student’s t-test with GraphPad. A minimum p value of *p* < 0.05 was considered as statistically significant.

## Figures and Tables

**Table 1:** Differentially expressed genes in HDAC5 vs. vector-only transduced UC cell lines.

Cell Lines	No. Genes FDR ^1^: *p* ≤ 0.05	No. Genes Fold Change ≥2	No. Genes Bonferroni: *p* ≤ 0.05	No. Genes Fold Change ≥2
RT112 Vector vs. HDAC5	3278	510 173 up 337 down	1245	261 69 up 192 down
VM-Cub-1 Vector vs. HDAC5	16,883	9387 3331 up 6056 down	12,074	6948 2645 up 4303 down
SW1710 Vector vs. HDAC5	10,941	2381 927 up 1454 down	5949	1265 516 up 749 down
UM-UC-3 Vector vs. HDAC5	8318	1379 542 up 837 down	4062	733 233 up 500 down

^1^ FDR: false discovery rate.

**Table 2:** Top canonical pathways in HDAC5 versus vector-only transduced UC cell lines.

Rank	RT112	VM-Cub-1	SW1710	UM-UC-3
1	LPS/IL-1 Mediated Inhibition of RXR Function (9.5% 21/221) ^1^	Axonal Guidance Signaling (46.9% 212/452)	Axonal Guidance Signaling (18.1% 82/452)	Hepatic Fibrosis/Hepatic Stellate Cell Activation (16.9% 31/183)
2	Granulocyte Adhesion and Diapedesis (10.1% 18/179)	Hepatic Fibrosis/Hepatic Stellate Cell Activation (55.2% 101/183)	LPS/IL-1 Mediated Inhibition of RXR Function (18.1% 40/221)	Axonal Guidance Signaling (10.8% 49/452)
3	Agranulocyte Adhesion and Diapedesis (9.4% 18/191)	Role of Macrophages, Fibroblasts and Endothelial Cells in Rheumatoid Arthritis (48.2% 150/311)	Coagulation System (34.3% 12/35)	Cellular Effects of Sildenafil (Viagra) (15.3% 20/131)
4	Xenobiotic Metabolism Signaling (7.3% 21/287)	Colorectal Cancer Metastasis Signaling (49.6% 123/248)	LXR/RXR Activation (20.7% 25/121)	Osteoarthritis Pathway (12.9% 27/210)
5	Inhibition of Matrix Metalloproteases (17.9% 7/39)	IL-8 Signaling (51.8% 102/197)	Hepatic Fibrosis/Hepatic Stellate Cell Activation (18.0% 33/183)	Coagulation System (25.7% 9/35)

^1^ Overlaps are given in % and number of hits of the total gene set.

**Table 3:** Top upstream regulators (and their predicted activation) in HDAC5 versus vector-only transduced UC cell lines.

Rank	RT112	VM-Cub-1	SW1710	UM-UC-3
1	TNF (Inhibited)	TNF (Inhibited)	TNF (Activated)	TNF (Inhibited)
2	SMARCA4 (not given)	estrogen receptor (Inhibited)	dexamethasone (not given)	Lipopolysaccharide (Inhibited)
3	Tretinoin (Inhibited)	TGFB1 (not given)	TGFB1 (Activated)	TGFB1 (not given)
4	Lipopolysaccharide (Inhibited)	SMARCA4 (Inhibited)	SMARCA4 (not given)	IFNG (Inhibited)
5	beta-estradiol (not given)	beta-estradiol (not given)	IFNG (not given)	IL1B (Inhibited)

## Supplementary Materials

Supplementary materials can be found at https://www.mdpi.com/1422-0067/20/9/2135/s1.

## Abbreviations

BCA	Bicinchoninic acid
DMEM	Dulbecco’s modified Eagle’s medium
EMT	Epithelial-mesenchymal transition
FDR	False discovery rate
HDAC	Histone deacetylase
IPA	Ingenuity Pathway analysis
SD	Standard deviation
TGF	Transforming growth factor
TNF	Tumor necrosis factor
UCC(s)	Urothelial carcinoma cell line(s)
