# Supplementary material for: HDAC5 Expression in Urothelial Carcinoma Cell Lines Inhibits Long-Term Proliferation but Can Promote Epithelial-to-Mesenchymal Transition
Source: Int J Mol Sci. 2019 Apr 30;20(9):2135. doi: 10.3390/ijms20092135 (PMC6539474; doi:10.3390/ijms20092135)
Supplement: Supplementary file 1 [file ijms-20-02135-s001.pdf]

Suppl. Fig. 1

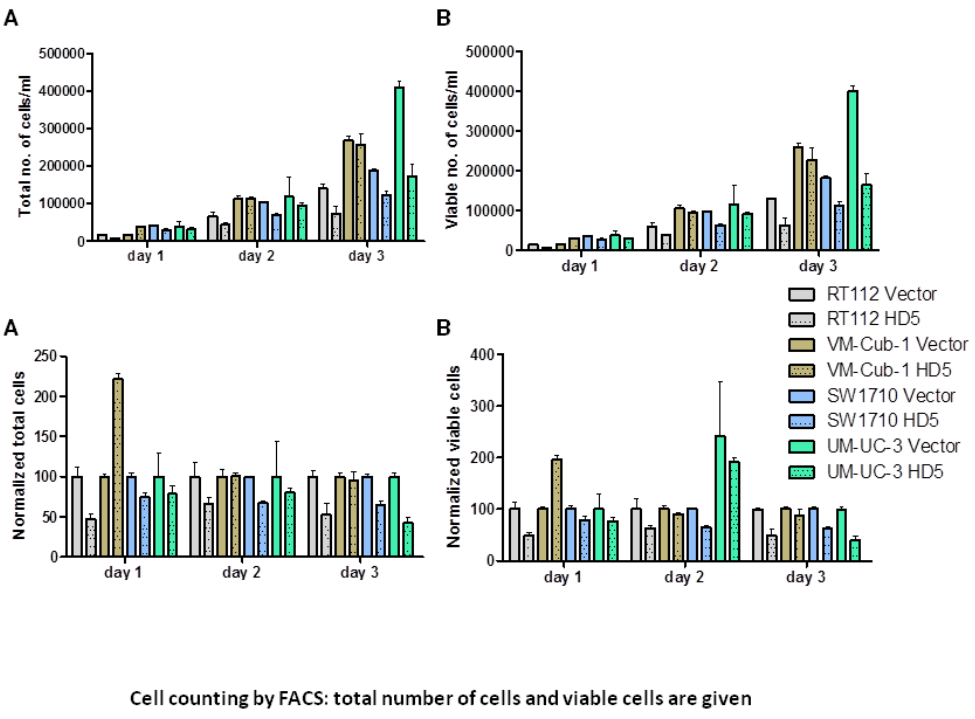

Figure S1: Proliferation of vector-only and HDAC5-transduced UCCs according to cell counting by FACS.

Suppl. Fig. 2

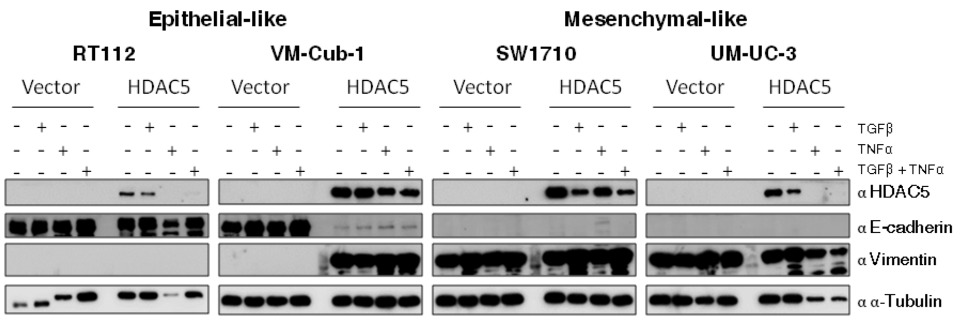

Figure S2: Immunoblot analysis of E-Cadherin and Vimentin expression following treatment with TGFβ or TNFα.
